# Central peptidergic modulation of peripheral olfactory responses

**DOI:** 10.1186/s12915-017-0374-6

**Published:** 2017-05-05

**Authors:** Sion Lee, Young-Joon Kim, Walton D. Jones

**Affiliations:** 1 Department of Biological Sciences, Korea Advanced Institute of Science and Technology (KAIST), Daejeon, South Korea; 2 School of Life Sciences, Gwangju Institute of Science and Technology (GIST), Gwangju, South Korea

**Keywords:** Insect olfaction, *Drosophila melanogaster*, Neuromodulation, Neuropeptide, NPF

## Abstract

**Background:**

Animal olfactory systems detect volatile environmental chemicals and integrate this information to direct the discovery of food and mates as well as danger avoidance. Rather than remaining constant, olfactory response thresholds are modulated by internal and external cues to adapt odor-guided behaviors to changing conditions.

**Results:**

Here, we show in *Drosophila melanogaster* that neuropeptide F (NPF) modulates the responses of a specific population of antennal olfactory sensory neurons (OSNs) to food-derived odors. We show that knock-down of NPF in NPF neurons specifically reduces the responses of the ab3A neurons to ethyl butyrate, a volatile ester found in apples and other fruits. Knock-down of the NPF receptor (NPFR) in the ab3A neuron reduces their responses and disrupts the ability of the flies to locate food. We also identify a sexual dimorphism in ab3A responsiveness: ab3A neurons in females immediately post-eclosion are less responsive to ethyl butyrate than those of both age-matched males and older females. Not only does this change correlate with brain NPF levels, but also NPFR mutants show no such sexual dimorphism. Finally, by way of mechanism, we show that mutation of NPFR seems to cause intracellular clustering of OR22a, the odorant receptor expressed in the ab3A neurons.

**Conclusions:**

Interestingly, this modulation of the peripheral odorant responsiveness of the ab3A neurons by NPF is distinct from the modulation of presynaptic gain in the ab3A neurons previously observed with the similarly named but distinct neuropeptide sNPF. Rather than affecting the strength of the output at the level of the first synapse in the antennal lobe, NPF-NPFR signaling may affect the process of odorant detection itself by causing intracellular OR clustering.

**Electronic supplementary material:**

The online version of this article (doi:10.1186/s12915-017-0374-6) contains supplementary material, which is available to authorized users.

## Background

Olfactory-guided behaviors, especially feeding behaviors, are central to both the positive and negative impacts insects have on human society. Honeybees and other insect pollinators depend heavily on their highly sensitive olfactory systems to find food. Similarly, blood-feeding insects like the malaria mosquito *Anopheles gambiae* would be much less effective in transmitting infectious pathogens if they lost their ability to detect the odor signatures of their human hosts.

The genetic model organism *Drosophila melanogaster* has two paired olfactory organs—the antennae and maxillary palps—covered in porous hair-like projections called sensilla [1]. These sensilla are filled with sensory lymph and house the dendrites of one to four olfactory sensory neurons (OSNs) [2]. Odor molecules diffuse through pores in the surfaces of the olfactory sensilla, dissolve in the sensory lymph, and are detected by odorant receptors (ORs) concentrated in the membranes of the OSN dendrites [3, 4]. Since insect ORs are ligand-gated ion channels, this odor binding gates a non-selective cation current that triggers action potentials in the OSNs [5, 6]. These odor-evoked spikes pass up the OSN axons bundled into the antennal nerve to the antennal lobe for processing before the olfactory information they encode passes to higher level processing centers like the mushroom body and lateral horn [7, 8].

Insect olfactory systems detect thousands of environmental chemicals at varying concentration thresholds for directing appropriate behavioral responses. These olfactory response thresholds, however, often require modulation depending on both internal and external cues to flexibly adapt odor-guided behaviors to changing environmental conditions. When an animal reaches sexual maturity, for example, an increase in its sensitivity to sex pheromones or other conspecific odors may improve its chances of productive mating [9–11].

Neuropeptides are a class of small protein-like neurotransmitters/neuromodulators secreted by specific populations of neurons to cause a broad range of neurophysiological effects [12, 13]. In hungry flies, elevated insulin levels increase the expression of the neuropeptide receptor sNPFR in food-related olfactory circuits (e.g., the OR42b OSNs), inducing a presynaptic facilitation that drives increased food search behaviors [14]. Elevated insulin also increases the expression of the tachykinin receptor DTKR, which reduces calcium responses in the OR85a antennal lobe glomerulus, which directs behavioral aversion [15]. The receptor for the neuropeptide CCHamide-1 was also recently implicated by Farhan et al. in the increased responses of OSNs expressing OR59b, OR7a, OR67d, or IR84a to their respective ligands [16].

Neuropeptide F (NPF) stimulates appetite like its mammalian homologue NPY [17]. Third instar *Drosophila* larvae lose their attraction to food and begin to wander about searching for a place to pupate. Overexpression of NPF can suppress sugar aversion in these larvae [18]. In hungry adult flies, increased levels of NPF enhance dopamine signaling, increasing the responses of the sugar-sensing GR5a gustatory receptor neurons (GRNs) [19]. The activity of the NPF neurons seems to signal the value of food odors to the animal, being tightly correlated with attraction behavior. Ablation of the four major NPF-producing cells in the brain disrupts behavioral attraction to the smell of yeast [20]. It remains unclear, however, whether NPF signaling plays any role in the OSNs themselves, especially in those that detect food odors.

Here, we identify a role for NPF in the response modulation of a specific population of OSNs in *Drosophila* that detect food-related fruity-smelling esters. We show that NPF neuron-specific knock-down of NPF reduces the responses of ab3A neurons, which express OR22a, to ethyl butyrate, a volatile ester found in apples and other fruits. We also show that OR22a neuron-specific knock-down of the NPF receptor NPFR produces a similar response reduction and disrupts the ability of flies to locate fruit odor baits. Thus, our results show that, rather than affecting presynaptic gain like the similarly named sNPF and sNPFR, NPF and NPFR seem to regulate the peripheral responses to food odors at the level of odorant detection.

## Results

### Modulation of peripheral olfactory responses to a food odor

The olfactory sensilla of *Drosophila* were first classified by morphology into basiconic, trichoid, and coeloconic shapes [21]. The largest are the club-shaped basiconic sensilla, which are divided by location, size, and OR expression into 13 subclasses [2]. Among the large antennal basiconics (ab), each sensillum of the ab3 class houses two OSNs designated ab3A and ab3B on the basis of spike amplitude, with ab3A giving the larger spikes [22]. These ab3A neurons express the olfactory co-receptor Orco along with the odorant-specific odorant receptor OR22a [23, 24]. OR22a responds to several fruity-smelling esters including ethyl butyrate, a volatile abundant in apples and many other fruits [25].

**Fig. 1:**
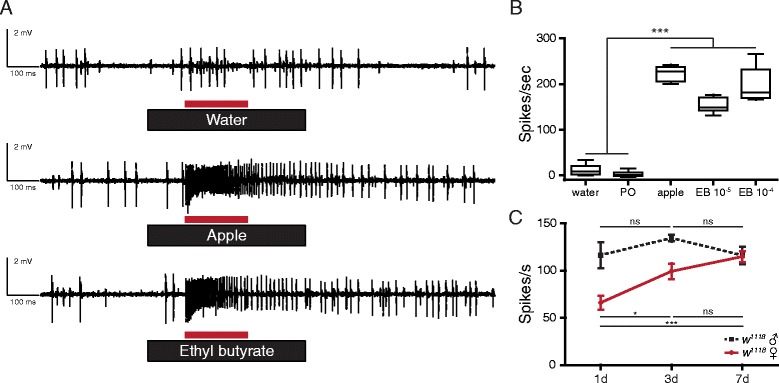
The responses of ab3A neurons vary with sex and maturity. **a** Sample traces for ab3 sensilla from multiple *w* ^*1118*^ male flies responding to a 0.5-s stimulation (*black bars*) of water, fresh apple odor, or ethyl butyrate (*EB*, 10^−5^ v/v). The *red bars* indicate the 200-ms quantification window. The larger spikes are the ab3A neurons, and the smaller spikes are the ab3B neurons. **b** Peak odor-evoked activity of the ab3A neurons of 7- to 14-day-old flies of both sexes (50:50) responding to water, paraffin oil (*PO*), fresh apple odor, EB (10^−5^ v/v), or EB (10^−4^ v/v). Boxplot whiskers indicate minimum and maximum values, one-way analysis of variance (*ANOVA*), *P* ≤ 0.001 (***). Replicate numbers for water = 15; paraffin oil = 16; apple = 6; EB 10^−5^ v/v = 10; EB 10^−4^ v/v = 9. **c** Peak odor-evoked responses of ab3A neurons of male (♂) and female (♀) *w* ^*1118*^ flies to ethyl butyrate (EB, 10^−5^ v/v) recorded 1, 3, and 7 days (*1d*, *3d*, *7d*) post-eclosion. The data are presented as means ± standard error, one-way ANOVA, non-significant (*ns*), *P* ≤ 0.05 (*), *P* ≤ 0.001 (***). Replicate numbers for 1d males = 6; 3d males = 7; 7d males = 8; 1d females = 6; 3d females = 6; 7d females = 6

Using the single sensillum electrophysiological recording technique, we confirmed that ab3A neurons are robustly activated by fresh apple odor and by ethyl butyrate, one of apple odor’s major components (Fig. 1a, b). Since an animal’s attraction to food odor depends on physiological states like sleep, hunger, and even age, we wondered if the peripheral responses of the ab3A neurons may be modulated. Therefore, we measured the responses of ab3A neurons to ethyl butyrate (EB) after rearing flies under several conditions designed to change their internal physiological states. Under our conditions, we saw no significant changes in the dose-response relationship of the ab3A neurons to EB after rearing on protein-rich or carbohydrate-rich diets, or rearing at high (29 °C) and low (18 °C) temperatures; neither did we see changes with circadian rhythm (data not shown). We found, however, that the responses of the ab3A neurons change as female flies mature post-eclosion. While the ab3A neurons of male *w* ^*1118*^ flies show a consistent response rate from 1–7 days post-eclosion, female ab3A neurons show a consistent age-dependent increase in EB responses reaching male levels by 7 days post-eclosion (Fig. 1c).

### NPF-NPFR signaling sensitizes ab3A neurons

**Fig. 2:**
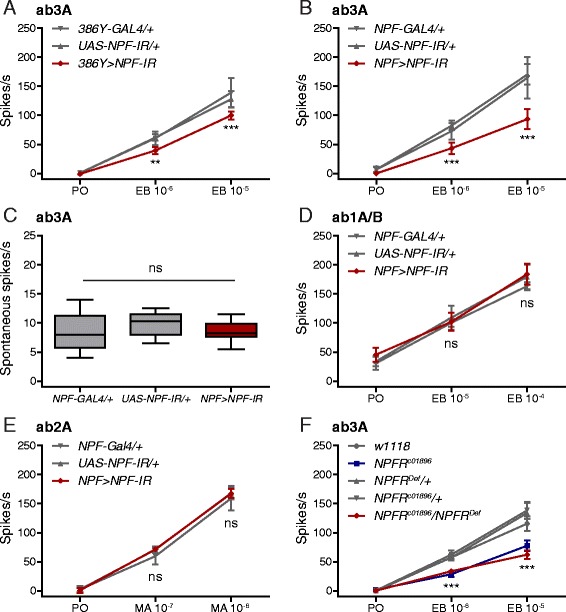
NPF-NPFR signaling sensitizes ab3A neurons. **a** Peak odor-evoked ab3A activity from *386Y > NPF-IR* flies and heterozygous controls responding to solvent (paraffin oil, *PO*) or ethyl butyrate (*EB* 10^−6^ or 10^−5^ v/v). Replicates: *386Y-GAL4/+*, PO = 14; EB 10^−6^ and 10^−5^ = 6; *UAS-NPF-IR/+*, PO = 25, EB 10^−6^ = 13, EB 10^−5^ = 12; *386Y > NPF-IR*, PO = 9, EB 10^−6^ and 10^−5^ = 6. **b** Peak odor-evoked ab3A activity from *NPF > NPF-IR* flies and heterozygous controls responding to PO or EB 10^−6^ or 10^−5^ v/v. *NPF-GAL4/+*, PO = 15; EB 10^−6^ and 10^−5^ = 6; *UAS-NPF-IR/+*, PO = 15, EB 10^−6^ and 10^−5^ = 6; *NPF > NPF-IR*, PO = 12, EB 10^−6^ and 10^−5^ = 6. **c** Spontaneous ab3A activity from *NPF > NPF-IR* flies and heterozygous controls. Boxplot whiskers indicate minimum and maximum values, *n* = 12, one-way ANOVA, non-significant (*ns*). **d** Peak odor-evoked ab1A/B activity from *NPF > NPF-IR* flies and heterozygous controls responding to PO or EB 10^−5^ or 10^−4^ v/v. Replicates for all genotypes: PO = 12, EB 10^−5^ and 10^−4^ = 6. **e** Peak odor-evoked ab2A activity from *NPF > NPF-IR* flies and heterozygous controls responding to PO or methyl acetate (*MA* 10^−7^ or 10^−6^ v/v). Replicates for all genotypes: PO = 12, EB 10^−7^ and 10^−6^ = 6. **f** Peak odor-evoked ab3A activity from *w* ^*1118*^, homozygous *NPFR* ^*c01896*^, transheterozygous *NPFR* ^*c01896*^ */NPFR* ^*Def*^, and heterozygous controls responding to PO or ethyl butyrate (EB 10^−6^ or 10^−5^ v/v). Replicates: *w* ^*1118*^, PO = 28, EB 10^−6^ and 10^−5^ = 14; *NPFR* ^*c01896*^, PO = 26, EB 10^−6^ and 10^−5^ = 12; *NPFR* ^*Def*^, PO = 15, EB 10^−6^ and 10^−5^ = 6; *NPFR* ^*c01896*^ */+*, PO = 17, EB 10^−6^ and 10^−5^ = 6; *NPFR* ^*c01896*^ */NPFR* ^*Def*^, PO = 15, EB 10^−6^ and 10^−5^ = 6. The data in **a**–**f** represent recordings from 7- to 14-day-old flies of both sexes (50:50). All heterozygous controls were crossed to ^*w1118*^. The data in **a**, **b**, and **d**–**f** are presented as means ± 95% confidence intervals, two-way ANOVA, non-significant (ns), *P* ≤ 0.01 (**), *P* ≤ 0.001 (***)

We expected that this modulation of the ab3A neurons may be peptidergic. The combination of a GAL4 enhancer trap expressed in many adult peptidergic neurons (*386Y-GAL4*) [26] with an RNA interference (RNAi) line specific to neuropeptide F (NPF) (*UAS-NPF-IR*) significantly reduces the responses of ab3A neurons to EB (Fig. 2a). In *Drosophila*, NPF affects several physiological phenomena including appetite [18] and the modulation of gustatory responses to sugar [19]. We also found that limiting the knock-down of NPF to NPFergic neurons using *NPF-GAL4* produces an even larger reduction in ab3A responses to EB (Fig. 2b). *NPF > NPF-IR* brains show almost no NPF signal compared to the appropriate heterozygous controls (i.e., crossed to *w* ^*1118*^) (Additional file 1: Figure S1A). Using two copies of NPF-GAL4 and two copies of the membrane-tethered Green fluorescent protein (GFP) transgene UAS-myr::GFP, we were able to visualize the projections of these cells into the antennal lobes (Additional file 1: Figure S1B, C). The reduction in peripheral olfactory responses is specific to the odor-evoked activity of the ab3A neurons, as their spontaneous activity is unaffected by NPF knock-down (Fig. 2c). It also seems to be specific to the ab3A neuronal subclass. The responses of both ab1A/B and ab2A to EB and methyl acetate (MA), respectively, are unaffected by NPF knock-down (Fig. 2d, e). Here, we did not sort ab1A spikes from ab1B spikes because the similar amplitudes of both classes of spikes complicate the sorting process. Although both ab1A and ab1B neurons respond to EB, because ab1A responses to EB are much stronger than ab1B responses, ab1A activity likely dominates the results shown in Fig. 2d. Although the ab2B, ab8A/B, and pb1A (palp basiconic 1A) sensilla also respond to EB, NPF knock-down does not affect them (Additional file 2: Figure S2).

Since NPF acts via the G protein-coupled NPF receptor (NPFR) [27], we next asked whether loss of NPFR also affects the responses of ab3A neurons to EB. The ab3A neurons of flies carrying homozygous piggyBac element insertions in the *NPFR* locus (*NPFR* ^*c01896*^) show significantly lower responses to EB than those of the control *w* ^*1118*^ genetic background (Fig. 2f). Although Krashes et al. verified *NPFR* ^*c01896*^ as an NPFR hypomorph [17], for further evidence of NPFR’s involvement, we combined *NPFR* ^*c01896*^ with a chromosomal deficiency covering the *NPFR* locus (*NPFR* ^*Def*^). As expected, transheterozygous loss of NPFR (*NPFR* ^*c01896*^ */NPFR* ^*Def*^) produces the same dramatic reduction in ab3A responses to EB compared to the corresponding heterozygous controls (Fig. 2f). We also found that *NPFR* ^*c01896*^ ab3A neurons show reduced responses to other apple odors including methyl butyrate (Additional file 3: Figure S3).

### NPF-NPFR signaling sensitizes ab3A neurons as flies mature

**Fig. 3:**
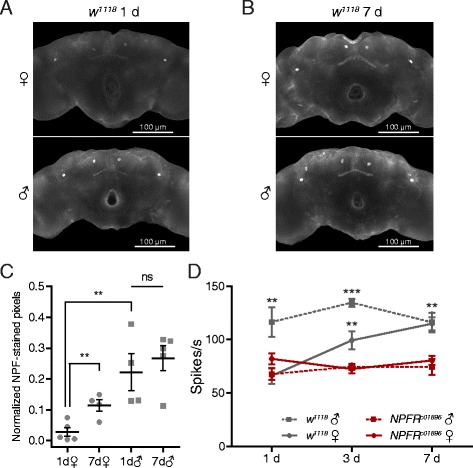
Sexual dimorphic NPF-NPFR signaling may account for the difference in ab3A responses of young males and females. **a**, **b** Representative images of adult male (♂) and female (♀) brains stained 1 (**a**) and 7 (**b**) days post-eclosion with an NPF-specific antiserum. **c** A pixel-based quantification of the NPF staining of male (♂) and female (♀) *w* ^*1118*^ brains 1 and 7 days post-eclosion. Maximal intensity projections of z-stacks comprising ten 5.1-μm-thick optical sections centered on the four largest and brightest NPF-producing cells were used for quantification. Mean number of pixels with intensity scores >100 from brains for each sex are normalized with the nc82 signal and presented ± standard error, Student’s *t* test, non-significant (*ns*), *P* ≤ 0.01 (**). Replicate numbers for 1d females = 5; 7d females = 4; 1d males = 4; 7d males = 5. **d** Peak odor-evoked activity of ab3A neurons from male (♂) and female (♀) *NPFR* ^*c01896*^ and *w* ^*1118*^ flies to ethyl butyrate (*EB*, 10^−5^ v/v) recorded 1, 3, and 7 days post-eclosion. Data are presented as means ± standard error, two-way ANOVA, *P* ≤ 0.01 (**), *P* ≤ 0.001 (***). Replicate numbers for 1d *w* ^*1118*^ males = 6; 3d *w* ^*1118*^ males = 7; 7d *w* ^*1118*^ males = 8; 1d *w* ^*1118*^ females = 6; 3d *w* ^*1118*^ females = 6; 7d *w* ^*1118*^ females = 6; 1d *NPFR* ^*c01896*^ males = 6; 3d *NPFR* ^*c01896*^ males = 6; 7d *NPFR* ^*c01896*^ males = 6; 1d *NPFR* ^*c01896*^ females = 6; 3d *NPFR* ^*c01896*^ females = 6; 7d *NPFR* ^*c01896*^ females = 6

Since the ab3A neurons of male and female flies (*w* ^*1118*^) show differences in their responses to ethyl butyrate 1 day after eclosion (Fig. 1c), and adult male brains have more NPF neurons than female brains of unspecified ages [28], we wondered whether the olfactory sexual dimorphism we identified can be attributed to NPF signaling. After dissecting brains from *w* ^*1118*^ males and females 1 and 7 days after eclosion, we stained them with an NPF-specific antiserum (Fig. 3a, b). Then, by processing similar stacks of confocal images of the brains from each sex with ImageJ [29], we found higher NPF staining per pixel in male brains than in female brains (Fig. 3c). We next compared the electrophysiological responses of ab3A neurons from *w* ^*1118*^ flies with those of *NPFR* ^*c01896*^ flies over the first week of adult life. We found that the ab3A neurons of both male (♂) and female (♀) *NPFR* ^*c01896*^ flies show low responses to ethyl butyrate (EB, 10^−5^ v/v) steadily through the first week post-eclosion (Fig. 3d). By comparison, the responses of *w* ^*1118*^ males are significantly higher from days 1–7. The responses of female *w* ^*1118*^ ab3A neurons, in contrast, are as low as those of *NPFR* ^*c01896*^ ab3A neurons on day 1, but rise to the level of male *w* ^*1118*^ ab3A neurons by day 7 (Fig. 3d). Note that the *w* ^*1118*^ data presented in Fig. 3d are identical to those in Fig. 1c and are shown again for comparison’s sake. Together, these results suggest that sexual dimorphisms in NPF signaling may account for the differential responsiveness of female ab3A neurons as they mature.

### ab3A neurons require NPFR to produce wild-type responses

**Fig. 4:**
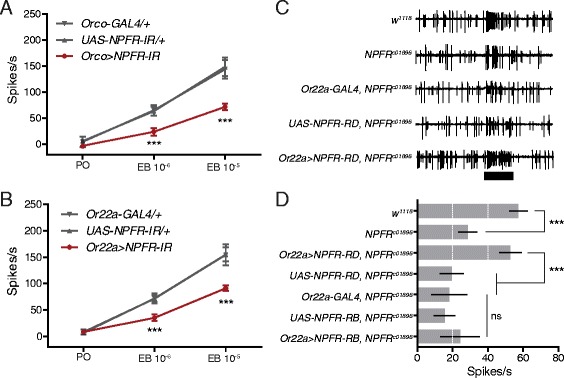
ab3A neurons express NPFR. **a** Peak odor-evoked ab3A activity from *Orco > NPF-IR* flies and heterozygous controls responding to solvent (paraffin oil, *PO*) or ethyl butyrate (*EB* 10^−6^ or 10^−5^ v/v). Replicates: *Orco-GAL4/+*, *n* = 6; *UAS-NPFR-IR/+*, PO = 8, EB 10^−6^ and 10^−5^ = 6; *Orco > NPFR-IR*, *n* = 6. **b** Peak odor-evoked ab3A activity from *Or22a > NPF-IR* flies and heterozygous controls responding to PO or EB 10^−6^ or 10^−5^ v/v. Replicates: *Or22a-GAL4/+*, PO = 12, EB 10^−6^ and 10^−5^ = 6; *UAS-NPFR-IR/+*, PO = 16, EB 10^−6^ and 10^−5^ = 10; *Or22a > NPFR-IR*, PO = 12, EB 10^−6^ and 10^−5^ = 6. The data in **a** and **b** are presented as means ± 95% confidence intervals, two-way ANOVA, *P* ≤ 0.001 (***). The heterozygous controls in **a** and **b** were crossed to ^*w1118*^. **c** Sample traces for ab3 sensilla from the indicated genotypes responding to a 0.5-s stimulation (*black bar*) of EB (10^−6^ v/v). **d** A quantification of the results presented in **c**. Recordings were performed on 7- to 14-day-old flies of both sexes. Ab3A neuron-specific expression of *NPFR-RD* but not *NPFR-RB* using *Or22a-GAL4* rescues the reduced EB responsiveness of the *NPFR* ^*c01896*^ mutant. The data in **d** are presented as means ± 95% confidence intervals, one-way ANOVA, *P* ≤ 0.001 (***). Replicate numbers for *w* ^*1118*^ = 14; *NPFR* ^*c01896*^ = 12; *Or22a > NPFR-RD, NPFR* ^*c01896*^ = 12; *UAS-NPFR-RD, NPFR* ^*c01896*^ = 12; *Or22a-GAL4, NPFR* ^*c01896*^ = 14; *UAS-NPFR-RB, NPFR* ^*c01896*^ = 12; *Or22a > NPFR-RB, NPFR* ^*c01896*^ = 12

We next asked where NPFR is required for determining ab3A responses. Since the most obvious candidate is the OSNs themselves, we first used *Orco-GAL4* to knock down NPFR in all the OSNs that express the olfactory co-receptor Orco. We found that ab3A neurons from *Orco > NPFR-IR* flies show significantly lower responses to ethyl butyrate (EB, 10^−6^ and 10^−5^ v/v) than their heterozygous controls (crossed to *w* ^*1118*^) (Fig. 4a). While *Orco-GAL4* labels most OSNs in the antennae and all OSNs in the maxillary palps, *Or22a-GAL4* labels only ab3A neurons. As with *Orco > NPFR-IR* flies, the ab3A neurons of *Or22a > NPFR-IR* flies show lower ab3A responses to EB than their heterozygous controls (Fig. 4b). Interestingly, we also found that knock-down of NPF in most OSNs with *Orco-GAL4* and ab3A neurons with *Or22a-GAL4* also produces a mild reduction in the responses of ab3A neurons to EB (Additional file 4: Figure S4). This suggests that the OSNs themselves may produce small amounts of NPF.

We next isolated total RNA from *w* ^*1118*^ heads for cDNA synthesis. From the resulting head cDNAs, we cloned versions of two of the NPFR transcripts annotated in Flybase, *NPFR-RB* and *NPFR-RD*. We used both to generate transgenic UAS-NPFR lines and then used them to perform an ab3A neuron-specific rescue of the *NPFR* ^*c01896*^ mutation. From the sample traces for this experiment, it is clear that *NPFR-RD* fully rescues the responses of ab3A neurons to EB (10^−6^ v/v) (Fig. 4c). In Fig. 4d, we present a quantification of recordings comparing the *w^1118* genetic background control to the *NPFR* ^*c01896*^ mutant and the ab3A neuron-specific rescue (*Or22a-GAL4 > UAS-NPFR-RD, NPFR* ^*c01896*^) compared to its appropriate heterozygous controls (Fig. 4d). Interestingly, although the *NPFR-RB* isoform appeared far more often among the *w* ^*1118*^ cDNAs we used to generate the UAS-NPFR lines than the *NPFR-RD* isoform, it fails to rescue the ab3A response phenotype (Fig. 4d). We speculate that the 23 C-terminal amino acid residues (382–404) of the NPFR-RD isoform absent in the NPFR-RB isoform may affect receptor trafficking, post-translational modifications, or receptor membrane topology (Additional file 5: Figure S5).

### NPFR in ab3A neurons modulates olfactory-guided behavior

**Fig. 5:**
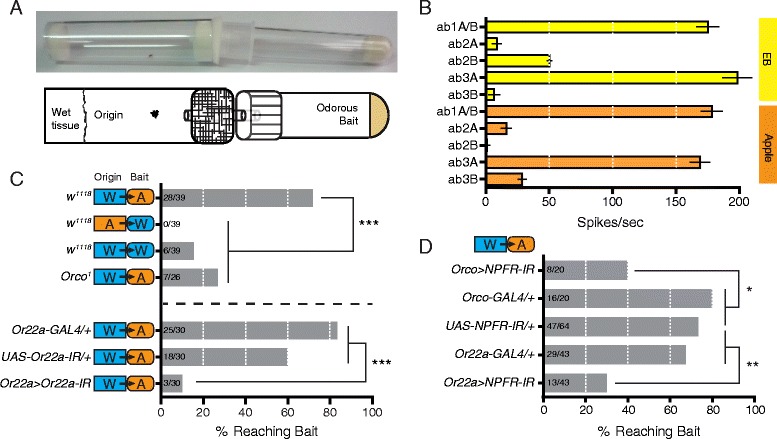
NPFR in ab3A neurons modulates olfactory-guided behavior. **a** Photo and schematic of the single fly olfactory trap assay. **b** Ethyl butyrate (10^−4^ v/v) and fresh apple juice (10^−1^ v/v) induce very similar electrophysiological responses in the OSNs of the large antennal basiconic sensilla ab1–ab3. Odor-evoked responses are presented as means ± standard error. Replicate numbers for EB - ab1A/B = 7; EB - ab2A = 7; EB - ab2B = 7; EB - ab3A = 9; EB - ab3B = 6; Apple - ab1A/B = 8; Apple - ab2A = 8; Apple - ab2B = 8; Apple - ab3A = 8; Apple - ab3B = 8. **c** Control experiments validating the single fly olfactory trap assay. *w* ^*1118*^ flies are robustly attracted to fresh apple juice, but not to water. *Orco* ^*1*^ mutants, which have defective OR trafficking and severely disrupted odor detection, show only slightly higher attraction to the apple juice bait (*A*) than *w* ^*1118*^ flies show to water (*W*). Ab3A neuron-specific knock-down of Or22a disrupts attraction to apple juice, as *Or22a > Or22a-IR* flies are much less successful finding the bait than heterozygous controls (crossed to *w* ^*1118*^). Replicate numbers for *w* ^*1118*^ W to A = 39; *w* ^*1118*^ A to W = 39; *w* ^*1118*^ W to W = 39; *Orco* ^*1*^ W to A = 26; *Or22a-GAL4/+* W to A = 30; *UAS-Or22a-IR/+* W to A = 30; *Or22a > Or22a-IR* W to A = 30. **d** Knock-down of NPFR in Orco-positive neurons (*Orco-Gal4*) and in ab3A neurons (*Or22a-Gal4*) significantly reduces attraction to apple juice. The data in **c** and **d** are presented as the percentage of flies reaching the bait (i.e., number of successes/number of single fly assays); *P* values were determined with Fisher’s exact test, *P* ≤ 0.05 (*), *P* ≤ 0.01 (**), *P* ≤ 0.001 (***). Replicate numbers for *Orco > NPFR-IR* = 20; *Orco-GAL4/+* = 20; *UAS-NPFR-IR/+* = 64; *Or22a-GAL4/+* = 43; *Or22a > NPFR-IR* = 43

We next asked whether NPF-NPFR signaling in the ab3A neurons modulates olfactory-guided behaviors. Trap assays test olfactory function at the behavioral level [30], but they sometimes produce noisy data (not shown). Since social cues modulate olfactory behavior in flies [31], we designed a new trap assay for individual flies (Fig. 5a), hoping to measure olfactory attraction at a higher signal-to-noise ratio. The single fly olfactory trap assay comprises an origin chamber and a bait chamber connected by a short piece of plastic tubing only slightly wider than an individual fly. Although the origin chambers contain wet tissue as a water source, they do not contain food. At first, the individual flies in the origin chamber resist traveling through the narrow tube, but as they starve over the course of the assay, the odor source lures them into the adjoining bait chamber. To perform the assay, we habituate individual 7- to 10-day-old male flies in vials containing a tissue soaked with fresh apple juice. After assembling the traps, we place them in a dark chamber at room temperature for 40 h. From 40–50 h, we check the traps every 2 h, removing and counting “successes” in which the individual flies travel to the bait chamber. We chose fresh apple juice (10^−1^ v/v) as a bait because it provides a source of sugar and induces a similar olfactory response in the OSNs of large basiconic sensilla as ethyl butyrate (10^−4^ v/v) (Fig. 5b).

We began by performing initial control experiments to confirm that our single fly trap assay measures olfactory attraction. We found that more than 70% of *w* ^*1118*^ flies successfully reach bait chambers containing apple juice, but they never leave the origin chamber when it contains the apple juice rather than the bait chamber (Fig. 5c). If both chambers contain water, fewer than 20% of *w* ^*1118*^ flies cross to the bait chamber within 50 h. Since *Orco* ^*1*^ flies lack the olfactory co-receptor Orco, which is required for OR trafficking, their OR-expressing OSNs are non-functional [23]. We found that fewer than 30% of *Orco* ^*1*^ flies locate the apple juice bait. These results validate the single fly trap assay for measuring olfactory-guided attraction to fruit odor.

Since ethyl butyrate is one of the major components of apple odor, we next asked whether OR22a—the odorant receptor expressed in the ethyl butyrate-sensing ab3A neurons—is required for behavioral attraction to apple juice. We found that ab3A-specific knock-down of OR22a using *Or22a-GAL4* dramatically reduces attraction to apple juice in the trap assay (Fig. 5c). This result suggested that NPF-NPFR signaling may also modulate behavioral attraction to apple odor. Since knock-down of NPF in the NPF neurons could alter the internal physiological state of the flies, making them feel more satiated and less likely to move to the odor bait chamber, we decided to limit our behavioral experiments to genetic manipulations of the OSNs. Knock-down of NPFR in most olfactory neurons using *Orco-GAL4* and knock-down in the ab3A neurons alone using *Or22a-GAL4* both significantly reduce success in locating the apple juice bait (Fig. 5d). Together, these results suggest that the role of NPF-NPFR signaling in modulating the peripheral responses of the ab3A neurons is behaviorally relevant for olfactory-guided foraging.

### Exploring the mechanism of NPFR-mediated olfactory modulation

**Fig. 6:**
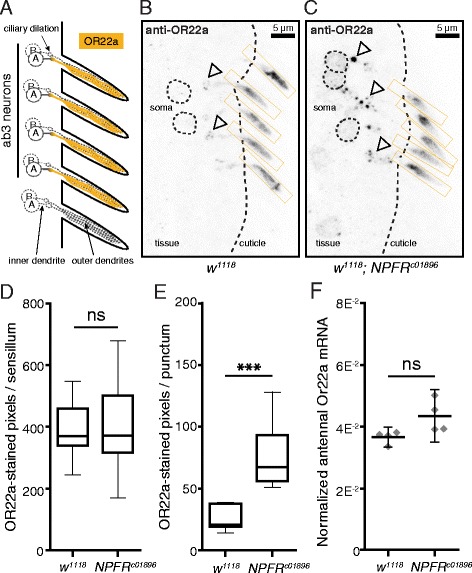
NPFR may affect OR22a localization but not expression. **a** A schematic showing the ab3A neurons’ soma, inner dendrites, ciliary dilations, and outer dendrites. Wild-type OR22a staining is indicated in *yellow*. **b**, **c** Representative images of ab3A neurons from *w* ^*1118*^ (*center*) and *NPFR* ^*c01896*^ (*right*) stained with an OR22a protein-specific antiserum. Some of the ciliary dilations are marked by *open triangles*. The color in these images is inverted to improve visibility. Scale bar, 5 μm. **d** Cropping only the outer dendritic segments as regions of interest (*yellow boxes*) and quantifying and selecting the pixels with intensity scores >50 using ImageJ, we found no difference between *w* ^*1118*^ and *NPFR* ^*c01896*^ ab3A neurons in OR22a staining. Boxplot whiskers indicate minimum and maximum values, *n* = 19 sensilla per genotype, Student’s *t* test, non-significant (*ns*). **e** By tightly cropping the puncta with the strongest signal intensity in each section, we found a significant increase in punctal staining near the ciliary dilations of *NPFR* ^*c01896*^ antennae as compared to *w* ^*1118*^ antennae. Boxplot whiskers indicate minimum and maximum values, Student’s *t* test, *P* ≤ 0.001 (***). Replicate numbers for *w* ^*1118*^ = 11; *NPFR* ^*c01896*^ = 14 puncta. **f** qPCR results show no significant difference in Or22a mRNA expression between *w* ^*1118*^ and *NPFR* ^*c01896*^ antennae of mixed sexes. Data are presented as means ± 95% confidence intervals, *n* = 4, Student’s *t* test, non-significant (ns)

A small swelling known as the ciliary dilation divides OSN dendrites into inner and outer segments (Fig. 6a). OSNs can only respond to odor by expressing and trafficking ORs to their outer dendritic segments where they can encounter odorant molecules [23, 32]. We hypothesized that NPF-NPFR signaling may modulate the responses of ab3A neurons to ethyl butyrate by altering either the expression or trafficking of the OR expressed in ab3A neurons that responds to ethyl butyrate, OR22a [24]. We, therefore, used an OR22a-specific antiserum to compare the levels of OR22a in the outer dendrites of ab3A neurons from *w* ^*1118*^ and *NPFR* ^*c01896*^ antennae. In *w* ^*1118*^ antennae, the ab3A neurons show almost no OR22a staining in the soma, little in the ciliary dilations, and a strong signal in the outer dendritic segments (Fig. 6b, Additional file 6: Figure S6). *NPFR* ^*c01896*^ mutant ab3A neurons show slightly stronger OR22a staining in their soma, strong punctate staining in their inner dendritic segments and ciliary dilations, and strong staining in their outer dendritic segments (Fig. 6c, Additional file 6: Figure S6). Except for the OR22a signal in the outer dendrites, this staining pattern in *NPFR* ^*c01896*^ ab3A neurons resembles that of *Orco* ^*1*^ mutant ab3A neurons, which lack the olfactory co-receptor Orco and hence show no OR22a in the outer dendrites [33]. Although this increase in intracellular OR22a in the absence of NPFR suggests a role for NPFR in modulating the dendritic trafficking or internalization of OR22a, we could not detect a difference when we quantified the OR22a-stained pixels in the outer dendrites of *w* ^*1118*^ and *NPFR* ^*c01896*^ antennae using ImageJ (Fig. 6d). Using a similar method, we did observe increased punctate staining near the ciliary dilations in *NPFR* ^*c01896*^ antennae compared to *w* ^*1118*^ antennae (Fig. 6e). Importantly, we were unable to detect any difference in *Or22a* mRNA levels between *w* ^*1118*^ and *NPFR* ^*c01896*^ antennae via qPCR (Fig. 6f), suggesting that the difference in OR22a protein staining in these puncta cannot be attributed to a change in Or22a expression.

## Discussion

Here, we identified a role in *Drosophila* for neuropeptide F (NPF) and its receptor NPFR in modulating the peripheral responses of the ab3A class of olfactory sensory neurons (OSNs). These neurons detect a range of fruity-smelling esters associated with the fruits that provide *Drosophila* food and a place to lay their eggs. We showed that loss of NPF in NPF neurons reduces odor-evoked spiking of ab3A neurons in response to apple odors (e.g., ethyl butyrate and methyl butyrate) without affecting their spontaneous activity. It does not, however, affect the responses of ab1A/B or ab2A neurons to their preferred ligands or several other neuron classes to ethyl butyrate (i.e., ab2B, ab8A/B, and pb1A). We showed that ab3A neurons must express NPFR themselves to exhibit this increase in olfactory responsiveness; ab3A-specific expression of NPFR rescues the reduced ab3A responses of *NPFR* ^*c01896*^ mutant flies. This modulation of olfactory responses by NPF and NPFR also affects olfactory-guided behaviors, as ab3A neuron-specific knock-down of NPFR reduces attraction of flies to apple juice baits.

Root et al. found that hunger enhances the responses of DM1, DM2, and DM4—the antennal lobe glomeruli that receive input from ab1A, ab3A, and ab2A neurons, respectively. They found that these neurons produce sNPF, which when released, alters the calcium responses of their own presynaptic terminals. This acts as a sort of gain control, enhancing the activation of the corresponding second-order olfactory projection neurons. In the ab1A neurons, Root et al. unambiguously attributed this presynaptic gain control to the sNPF receptor sNPFR1 and showed that insulin signaling and starvation both alter sNPFR1 expression [14]. This means that the ab1A neurons, which respond to food-related esters like the ab3A neurons, induce larger responses in their associated projection neurons in times of starvation, enhancing foraging behavior. The NPF-mediated modulation of ab3A neurons we discovered seems to differ from this sNPF-mediated gain control. Rather than magnifying presynaptic calcium responses as sNPFR activation seems to do, NPFR activation increases the number of odor-evoked action potentials in the ab3A neurons. It is unclear how these two pathways relate and what other implications these distinctions may have, but they suggest that the modulation of ab3A neurons by NPF and sNPF act through different molecular mechanisms. It is distinct from NPF’s modulation of sugar taste detection, which was discovered by Inagaki et al. They found that hunger and NPF increase sugar responses indirectly by modulating the influence of upstream dopaminergic neurons on the GR5a-positive GRNs in the labellum [19].

Lin et al. recently reported evidence that juvenile hormone (JH), which is secreted into the circulating hemolymph by the corpora allata, acts on its receptor Methoprene-tolerant (Met) in the pheromone-sensitive antennal trichoid at4 neurons to sensitize them [34]. While hormones are secreted into the circulation to act on distant target tissues, neuropeptides are typically secreted from peptide-producing neurons onto neighboring cells [13]. No one had previously reported NPFergic innervation of the antennae or antennal lobes, so we initially suspected that NPF may be similarly secreted into the circulation. But by combining two copies of NPF-GAL4, we were able to visualize NPF-GAL4-positive innervation of the antennal lobes. Since we also found that knock-down of NPF in the ab3A neurons themselves reduces their responses to EB, NPF seems to be acting locally.

To address the mechanism by which NPF-NPFR signaling modulates ab3A neurons, we stained NPFR mutant antennae with an antiserum that recognizes OR22a, the odorant receptor expressed by the ab3A neurons. We could not detect a difference in OR22a staining in the outer ab3A dendrites where odorant binding takes place. Still, compared to control antennae, NPFR mutant antennae show dramatically more OR22a-positive puncta near the ciliary dilations that separate the inner and outer ab3A dendritic segments. It is unclear what these OR22a-positive puncta are, but we expect that they represent either OR22a molecules whose trafficking to the ciliated outer dendrites is being blocked or those whose internalization from the periphery is being enhanced. These puncta strongly resemble the OR22a-positive puncta that appear in the absence of the olfactory co-receptor Orco required for OR trafficking to the ciliated outer dendrites and co-localize with markers of the endoplasmic reticulum [33]. This could support the former hypothesis—a specific reduction in dendritic OR22a trafficking in the absence of NPFR—but the OR22a staining in the outer dendrites of *NPFR* ^*c01896*^ antennae seems to be unaffected.

Although the molecular dynamics of OR dendritic surface localization, odorant binding, internalization, deactivation, and recycling remain somewhat unclear, we speculate that NPFR may modulate OR recycling. If NPFR acts to stabilize active odorant/OR complexes at the cell surface, effectively delaying receptor inactivation, each odorant stimulus would elicit more action potentials. Reduced signaling through NPFR could accelerate the internalization of the active odorant/OR complexes, effectively reducing the length of time they spend on the outer dendritic membrane. If our speculation proves true, the wild-type levels of OR22a in the *NPFR* ^*c01896*^ mutant antennae suggest that there may be a compensatory increase in the trafficking of new OR complexes to the outer segment. In other words, if our speculation is true, there should be a tight coupling between OR externalization and internalization. Since NPFR typically inhibits adenylyl cyclase and reduces neuronal activity [27], it is unclear how such a modulation of OR recycling would occur. Future studies should address the precise mechanisms that guide the movement of ORs in and out of the outer dendrites and how the various peptidergic signaling pathways may modulate those movements.

In this study, we also found that female flies show lower ab3A responses immediately post-eclosion than male flies, but this difference disappears as the flies age. We found a clear correlation between ab3A responses and NPF staining in young female versus male brains and showed that this sexual dimorphism is absent in *NPFR* ^*c01896*^ mutants. Unfortunately, we were unable to directly compare males and females in the trap assay, especially when they are young. Young males and females have dramatically different levels of body fat and appetites. Because of this, females require much longer periods of starvation to motivate them to move through the trap than males. This is why we used only males for this behavioral assay and why we were only able to focus on the role of NPFR in the OSNs rather than on sex-specific differences in behavior. The function of this sexual dimorphism is unclear, but it may enhance dispersal of young females to new and more palatable food sources. Once *Drosophila* larvae reach their final larval instar, they stop foraging and move out of their food to find a dry location to pupate. A piece of fruit suitable for laying eggs before a single round of the 10- to 14-day *Drosophila* life cycle may be less suitable for a second. Thus, the reduction in olfactory responses to fruit odors we observe in young female flies may help encourage them to find new food sources for egg laying. It will be interesting to test this hypothesis in future studies.

## Conclusions

The majority of the impact insects have on human society stems from their feeding behaviors (e.g., destroying our crops or transmitting disease through infectious bites). Since insect feeding behaviors are guided by olfaction, we were interested in how insect olfactory systems change in response to internal and external cues. Here, we show in the genetic model insect *Drosophila melanogaster* that NPF acts on its receptor NPFR to sensitize a specific population of antennal olfactory neurons that detect an important food-related odorant. We show that this peripheral olfactory modulation by NPF and NPFR is sexually dimorphic in young adult flies and that it affects olfactory-guided attraction to food odors. Since homologues of NPF and NPFR exist across insect species, it will be interesting to see whether these homologues also modulate olfactory food detection in these species. If so, this modulation may represent another potential target for future pest control strategies.

## Methods

### Fly stocks

Unless otherwise indicated, we maintained all fly stocks at 25 °C and 60% relative humidity under a 12 h:12 h light:dark cycle on standard cornmeal-yeast-corn syrup medium with 1.5 g/L of the anti-fungal Tegosept. We acquired the following fly stocks from either the Bloomington *Drosophila* Stock Center (BDSC) or the Vienna *Drosophila* Resource Center (VDRC) for our experiments: *Canton-S* (BDSC #1), *w* ^*1118*^ (BDSC #5905), *NPF-GAL4* (BDSC #25681, #25682), *UAS-myr::GFP* (BDSC #32197, #32198), *UAS-NPF-IR* (VDRC #108772), *386Y-GAL4* (BDSC #25410), *NPFR* ^*c01896*^ (BDSC #10747), *NPFR* ^*Def*^ (BDSC #1982), *Orco-GAL4* (BDSC #23292), *Or22a-GAL4* (BDSC #9951), *UAS-NPFR-IR* (VDRC #9605). We generated the *UAS-NPFR-RD* and *UAS-NPFR-RB* stocks by cloning NPFR cDNAs from *Drosophila* heads, subcloning them into a pUAS-T attB-containing vector, and injecting the resulting plasmids into eggs of a fly stock containing an attP landing site (BDSC #9736) using standard techniques.

### Electrophysiology

We performed single sensillum electrophysiological recordings for 7- to 14-day-old flies by inserting electrolytically sharpened tungsten electrodes into large basiconic sensilla as previously described [35]. We used a universal single-ended probe, an IDAC4 data acquisition controller, a CS55 stimulus controller, and the Autospike software (Syntech, Kirchzarten, Germany).

We purchased all odors from Sigma-Aldrich (St. Louis, MO, USA) at the highest purity available, suspended them in paraffin oil at the indicated dilutions, and delivered them by adding 3 μl of odor-paraffin oil emulsion to a small piece of filter paper. These were immediately inserted into standard Pasteur pipettes. After connecting these odor cartridges in-line with the stimulus controller, we used a foot pedal to trigger 0.5-s switches between odorous and non-odorous air streams directed at the antennae of live, restrained flies.

We calculated peak odor-evoked firing rates by counting the spikes occurring in the most action potential-rich 200-ms stretch of time within a 0.5-s window after odor stimulation. After converting this to spikes per second, we subtracted the number of spontaneous action potentials that occurred within a 1-s window before odor delivery.

For electrophysiological recordings, we chose not to starve the flies to avoid confounding variables because starvation is already known to increase the sensitivity of the ab3A neurons [14].

### Quantitative PCR

We prepared complementary DNAs (cDNAs) for qPCR by extracting total RNA from *Drosophila* antennae and reverse transcribing them with oligo(dT)20 primers and the SuperScript III First-Strand Synthesis System (Invitrogen, USA). We then performed qPCR amplification using the TOPrealTM qPCR 2X PreMIX (Enzynomics, Daejeon, South Korea) and a CFX96TM Real-Time System (Bio-Rad, Hercules, CA, USA). We used RpL32 for normalization and quantified Or22a expression using the Bio-Rad CFX Manager 3.1 software package. The primers (5′ to 3′) were as follows: Or22a (CGACGAACAGTTTTACATCTC, AATGCGTCAACATAGTCCAA); RpL32 (GCTAAGCTGTCGCACAAATG, CAATCTCCTTGCGCTTCTTG).

### Immunofluorescence

We performed antennal immunostaining experiments as previously described [24] using immunopurified rabbit polyclonal antiserum raised against an OR22a peptide (NH_2_-C-MLSKFFPHIKEKPLSERVKS-COOH) [24]) generated by AbFrontier (Project ID: 22a-1, Seoul, South Korea) at 1.3 μg/ml. As a secondary antibody, we used goat anti-rabbit IgG Alexa Fluor 488 (ab150085, lot #GR197834-1, Abcam, Cambridge, UK) at 1:800 v/v.

Using the same confocal settings for all antennal sections, we generated maximal intensity projections comprising six 2.3-μm-thick optical sections. We then quantified OR22a staining in the outer dendrite and ciliary dilation regions by counting all pixels with intensity scores above 50 using the digital image processing program ImageJ [29]. We used rectangular regions of interest (ROIs) encompassing all the visible staining for each outer dendritic segment and for individual OR22a-positive puncta. Within each ROI, we used the Color Threshold function to automatically select pixels above a set brightness level (>50) and the Measure function to count them (Additional file 6: Figure S6).

After fixing whole fly bodies in 4% paraformaldehyde, we performed whole-mount brain immunostaining experiments as previously described [7] using a rabbit polyclonal NPF-specific antiserum (RB-19-0001, lot #1006009NPFP, RayBiotech, Norcross, GA, USA) at 0.1 mg/ml, a chicken polyclonal GFP-specific antiserum (ab13970, lot #GR53074-4, Abcam) at 2 μg/ml, and the monoclonal nc82 antibody (AB_2314866, Developmental Studies Hybridoma Bank (DSHB), Iowa City, IA, USA) at 7 μg/ml. As secondary antibodies, we used goat anti-rabbit IgG Alexa Fluor 488 (ab150085, lot #GR197834-1, Abcam) and goat anti-mouse IgG Alexa Fluor 594 (A-11005, Thermo Fisher Scientific, Waltham, MA, USA) at 1:100 v/v.

Using the same confocal settings for all brains, we generated maximal intensity projections of z-stacks comprising ten 5.1-μm-thick optical sections centered on the four largest and brightest NPF-producing cells. We then quantified NPF staining intensity by counting all pixels with intensity scores above 100 (brightness ≥100) and normalized to the reference nc82 neuropil marker channel using ImageJ.

### Behavior

For the behavioral assays, we aged male flies 7–10 days on standard food and then habituated them on apple juice for 1 day in the dark. For each genotype or condition, we then introduced individual flies (*n* ≥ 10) into origin vials connected to bait vials by a narrow length of straw just wide enough for a single fly to pass. Figure 5a shows a photo and a schematic of a completed olfactory trap assembly. After aging and habituation, we placed each loaded trap assembly in a dark chamber at room temperature for 40 h. From 40–50 h, we checked the assay assemblies every 2 h to remove those in which the flies had moved into the bait chambers. We used this strategy because preliminary experiments indicated the flies grow sufficiently hungry and therefore motivated to find food over the initial 40 hours of starvation. Although we did not starve the flies used for the electrophysiological recordings, the trap assay requires hunger for motivation. Therefore, we were careful to use only the closest genetic controls (i.e., Or22a > NPFR-IR with the heterozygous UAS-alone and GAL4-alone controls) under identical conditions. As the results for this assay are binary (i.e., success or failure in reaching the bait), we used Fisher’s exact test in our statistical analysis of the results.

## Additional files

Additional file 1: Figure S1. NPF expression patterns. **A** Brains from male flies >10 days post-eclosion stained with an NPF-specific antiserum (*green*) show clear NPF depletion in NPF-G4 > NPF-IR flies compared to the NPF-GAL4/+ and UAS-NPF-IR/+ heterozygous controls. All brains are counter-stained with the nc82 neuropil marker (*magenta*). Scale bars, 50 μm. **B**, **C** Brains from *(2x)NPF-Gal4 > (2x)UAS-myr::GFP* flies stained with a GFP-specific antiserum show neuronal processes of NPF neurons innervating the antennal lobes in both males and females (**C**). (PDF 6184 kb)

Additional file 2: Figure S2. NPFR loss of function does not affect other OSNs that respond to ethyl butyrate. **A** Peak odor-evoked activity of the ab2B neurons from *w* ^*1118*^ and *w* ^*1118*^ *; NPFR* ^*c01896*^ mutant flies responding to solvent (paraffin oil, *PO*) or ethyl butyrate (10^−5^ v/v, 10^−4^ v/v, 10^−3^ v/v). Replicate numbers for PO, *w* ^*1118*^ = 11; EB 10^−5^ v/v, *w* ^*1118*^ = 6; EB 10^−4^ v/v, *w* ^*1118*^ = 6; EB 10^−3^ v/v, *w* ^*1118*^ = 6; PO, *NPFR* ^*c01896*^ = 12; EB 10^−5^ v/v, *NPFR* ^*c01896*^ = 6; EB 10^−4^ v/v, *NPFR* ^*c01896*^ = 6; EB 10^−3^ v/v, *NPFR* ^*c01896*^ = 6. **B** Peak odor-evoked activity of the ab8A/B neurons from *w* ^*1118*^ and *w* ^*1118*^ *; NPFR* ^*c01896*^ mutant flies responding to solvent (paraffin oil, PO) or ethyl butyrate (EB 10^−6^, 10^−5^ v/v, 10^−4^ v/v). Replicate numbers for PO, *w* ^*1118*^ = 12; EB 10^−6^ v/v, *w* ^*1118*^ = 6; EB 10^−5^ v/v, *w* ^*1118*^ = 6; EB 10^−4^ v/v, *w* ^*1118*^ = 6; PO, *NPFR* ^*c01896*^ = 10; EB 10^−6^ v/v, *NPFR* ^*c01896*^ = 6; EB 10^−5^ v/v, *NPFR* ^*c01896*^ = 6; EB 10^−4^ v/v, *NPFR* ^*c01896*^ = 6. **C** Peak odor-evoked activity of the pb1A neurons from *w* ^*1118*^ and *w* ^*1118*^ *; NPFR* ^*c01896*^ mutant flies responding to solvent (paraffin oil, PO) or ethyl butyrate (EB 10^−6^, 10^−5^ v/v, 10^−4^ v/v). Replicate numbers for PO, *w* ^*1118*^ = 14; EB 10^−6^ v/v, *w* ^*1118*^ = 6; EB 10^−5^ v/v, *w* ^*1118*^ = 6; EB 10^−4^ v/v, *w* ^*1118*^ = 6; PO, *NPFR* ^*c01896*^ = 14; EB 10^−6^ v/v, *NPFR* ^*c01896*^ = 7; EB 10^−5^ v/v, *NPFR* ^*c01896*^ = 8; EB 10^−4^ v/v, *NPFR* ^*c01896*^ = 8. The data in A, B, and C represent recordings from 7- to 14-day-old flies of both sexes (50:50) presented as means ± 95% confidence intervals. Two-way analysis of variance (*ANOVA*), non-significant (*ns*). (PDF 1331 kb)

Additional file 3: Figure S3. NPFR loss of function reduces the responses of ab3A neurons to methyl butyrate and apple odor. **A** Peak odor-evoked activity of ab3A neurons from *w* ^*1118*^ and *w* ^*1118*^ *; NPFR* ^*c01896*^ mutant flies responding to solvent (paraffin oil, *PO*) or a major apple odor: methyl butyrate (*MB*, 10^−6^ v/v, 10^−5^ v/v, 10^−4^ v/v). The data in A represent recordings from 7- to 14-day-old flies of both sexes and are presented as means ± 95% confidence intervals. Two-way ANOVA, non-significant (*ns*), *P* < 0.01 (**), *P* < 0.001 (***). Replicate numbers for PO, *w* ^*1118*^ = 12; MB 10^−6^ v/v, *w* ^*1118*^ = 6; MB 10^−5^ v/v, *w* ^*1118*^ = 6; MB 10^−4^ v/v, *w* ^*1118*^ = 6; PO, *NPFR* ^*c01896*^ = 12; MB 10^−6^ v/v, *NPFR* ^*c01896*^ = 6; MB 10^−5^ v/v, *NPFR* ^*c01896*^ = 6; MB 10^−4^ v/v, *NPFR* ^*c01896*^ = 6. **B** Peak odor-evoked activity from ab3A neurons of 7- to 14-day-old flies of both sexes responding to apple skin odor. Boxplot whiskers indicate minimum and maximum values, one-way ANOVA, *P* < 0.001 (***). Replicate numbers for apple, *w* ^*1118*^ = 6; apple, *NPFR* ^*c01896*^ = 7. (PDF 1298 kb)

Additional file 4: Figure S4. OSN-specific knock-down of NPF also affects ab3A responses. **A** Peak odor-evoked activity of ab3A neurons from *Or22a > NPF-IR* flies and the appropriate heterozygous GAL4 and UAS controls (crossed to *w* ^*1118*^) responding to solvent (paraffin oil, *PO*) or ethyl butyrate (*EB* 10^−6^ or 10^−5^ v/v). Replicate numbers for PO, *Or22a-GAL4+* = 16; EB 10^−6^ v/v, *Or22a-GAL4/+* = 8; EB 10^−5^ v/v, *Or22a-GAL4/+* = 8; PO, *UAS-NPF-IR/+* = 12; EB 10^−6^ v/v, *UAS-NPF-IR/+* = 6; EB 10^−5^ v/v, *UAS-NPF-IR/+* = 6; PO, *Or22a > NPF-IR* = 12; EB 10^−6^ v/v, *Or22a > NPF-IR* = 6; EB 10^−5^ v/v, *Or22a > NPF-IR* = 6. **B** Peak odor-evoked activity of ab3A neurons from *Orco > NPF-IR* flies and the appropriate heterozygous GAL4 and UAS controls (crossed to *w* ^*1118*^) responding to solvent (paraffin oil, PO) or ethyl butyrate (EB 10^−6^ or 10^−5^ v/v). These data in A and B are presented as means ± 95% confidence intervals. Two-way ANOVA, *P* < 0.05 (*). Replicate numbers for PO, *Orco-GAL4/+* = 12; EB 10^−6^ v/v, *Orco-GAL4/+* = 6; EB 10^−5^ v/v, *Or22a-GAL4/+* = 6; PO, *UAS-NPF-IR/+* = 12; EB 10^−6^ v/v, *UAS-NPF-IR/+* = 6; EB 10^−5^ v/v, *UAS-NPF-IR/+* = 6; PO, *Or22a > NPF-IR* = 16; EB 10^−6^ v/v, *Or22a > NPF-IR* = 8; EB 10^−5^ v/v, *Or22a > NPF-IR* = 8. (PDF 1289 kb)

Additional file 5: Figure S5. Hydrophobicity plots for the various NPFR isoforms. Hydrophobicity plots and membrane topology predictions for the NPFR-RD, RA, RB, and RC isoforms. (PDF 1821 kb)

Additional file 6: Figure S6. Staining OR22a-positive puncta. **A** The seven puncta with the highest signal intensities from *w* ^*1118*^ and *NPFR* ^*c01896*^ antennal sections. **B** Examples of region of interest selection by ImageJ where only pixels with intensity scores above 50 are included (*yellow boundary*). (PDF 1353 kb).

Additional file 7: Data values for Figs. 3c and 6f. (XLSX 11 kb)

## Abbreviations

EB: Ethyl butyrate
JH: Juvenile hormone
NPF: Neuropeptide F
NPFR: NPF receptor
OR: Odorant receptor
OSN: Olfactory sensory neuron
sNPF: Short NPF
sNPFR: sNPF receptor
