# Supplementary figures and images for: Central peptidergic modulation of peripheral olfactory responses
Source: BMC Biol. 2017 May 5;15:35. doi: 10.1186/s12915-017-0374-6 (PMC5420127; doi:10.1186/s12915-017-0374-6)

A

anti-NPF

*NPF-GAL4/+*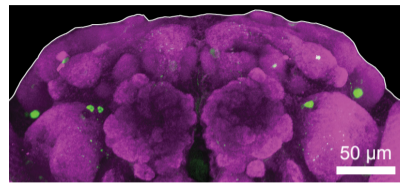*UAS-NPF-IR/+*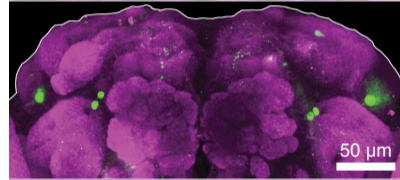*NPF>NPF-IR*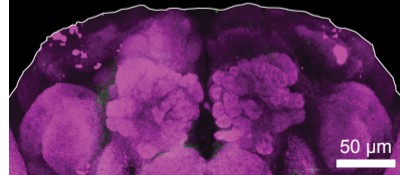

B

(2x)*NPF-Gal4*>(2x)*UAS-myr::GFP*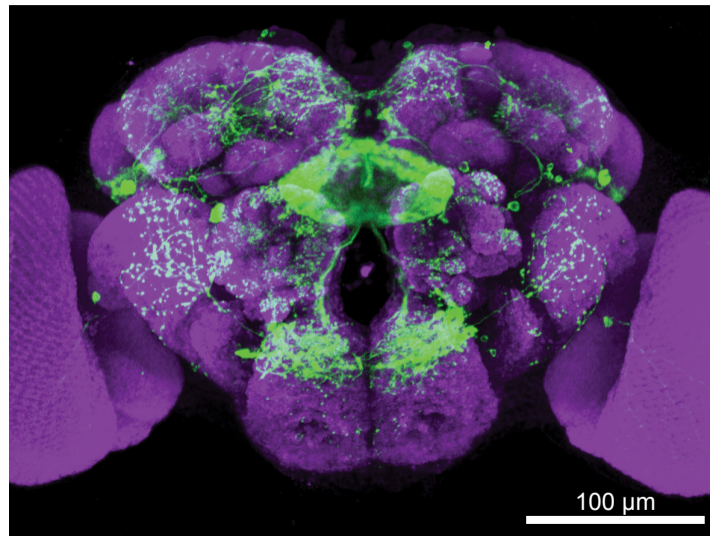

C

♀

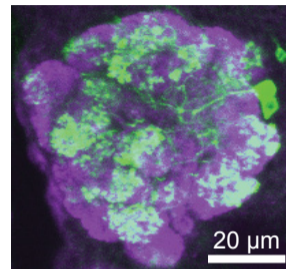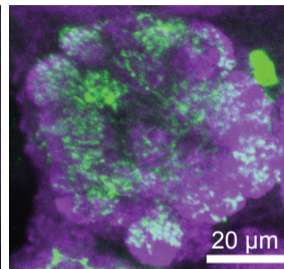

♂

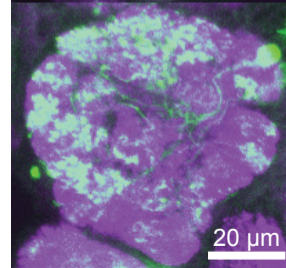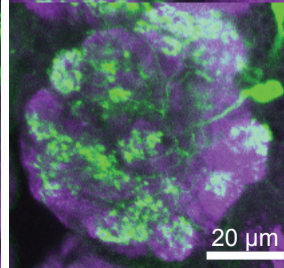

Supplement: Supplementary file 1 — NPF expression patterns. A Brains from male flies >10 days post-eclosion stained with an NPF-specific antiserum (green) show clear NPF depletion in NPF-G4 > NPF-IR flies compared to the NPF-GAL4/+ and UAS-NPF-IR/+ heterozygous controls. All brains are counter-stained with the nc82 neuropil marker (magenta). Scale bars, 50 μm. B, C Brains from (2x)NPF-Gal4 > (2x)UAS-myr::GFP flies stained with a GFP-specific antiserum show neuronal processes of NPF neurons innervating the antennal lobes in both males and females (C). (PDF 6184 kb) [file 12915_2017_374_MOESM1_ESM.pdf]

**A****ab2B (Or85a)**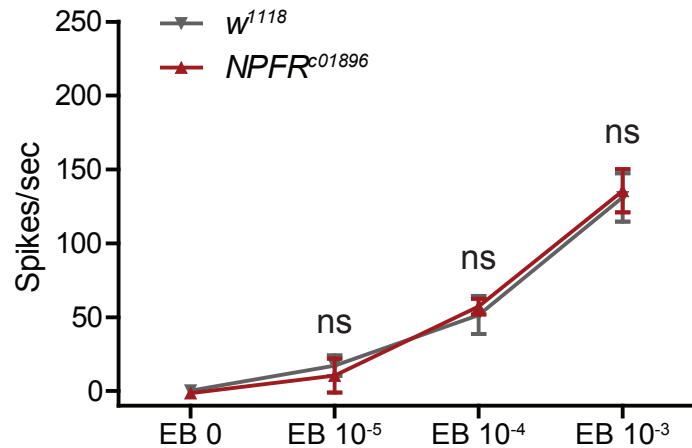**B****ab8A/B (Or43b/Or9a)**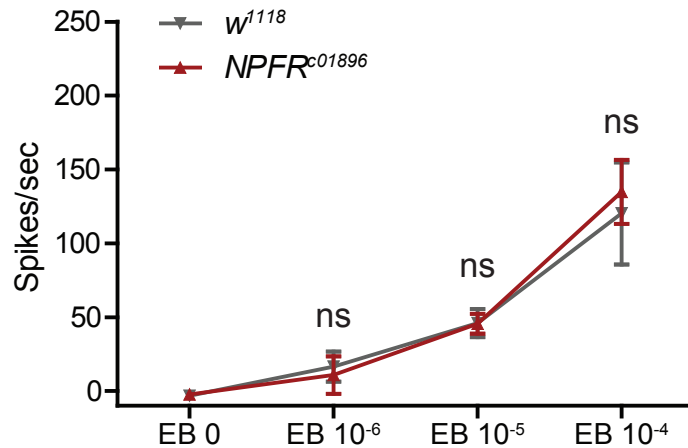**C****pb1A (Or42a)**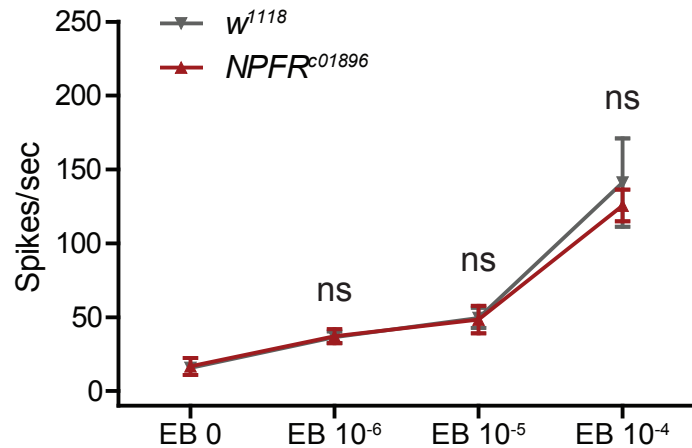

Supplement: Supplementary file 2 — NPFR loss of function does not affect other OSNs that respond to ethyl butyrate. A Peak odor-evoked activity of the ab2B neurons from w 1118 and w 1118 ; NPFR c01896 mutant flies responding to solvent (paraffin oil, PO) or ethyl butyrate (10−5 v/v, 10−4 v/v, 10−3 v/v). Replicate numbers for PO, w 1118 = 11; EB 10−5 v/v, w 1118 = 6; EB 10−4 v/v, w 1118 = 6; EB 10−3 v/v, w 1118 = 6; PO, NPFR c01896 = 12; EB 10−5 v/v, NPFR c01896 = 6; EB 10−4 v/v, NPFR c01896 = 6; EB 10−3 v/v, NPFR c01896 = 6. B Peak odor-evoked activity of the ab8A/B neurons from w 1118 and w 1118 ; NPFR c01896 mutant flies responding to solvent (paraffin oil, PO) or ethyl butyrate (EB 10−6, 10−5 v/v, 10−4 v/v). Replicate numbers for PO, w 1118 = 12; EB 10−6 v/v, w 1118 = 6; EB 10−5 v/v, w 1118 = 6; EB 10−4 v/v, w 1118 = 6; PO, NPFR c01896 = 10; EB 10−6 v/v, NPFR c01896 = 6; EB 10−5 v/v, NPFR c01896 = 6; EB 10−4 v/v, NPFR c01896 = 6. C Peak odor-evoked activity of the pb1A neurons from w 1118 and w 1118 ; NPFR c01896 mutant flies responding to solvent (paraffin oil, PO) or ethyl butyrate (EB 10−6, 10−5 v/v, 10−4 v/v). Replicate numbers for PO, w 1118 = 14; EB 10−6 v/v, w 1118 = 6; EB 10−5 v/v, w 1118 = 6; EB 10−4 v/v, w 1118 = 6; PO, NPFR c01896 = 14; EB 10−6 v/v, NPFR c01896 = 7; EB 10−5 v/v, NPFR c01896 = 8; EB 10−4 v/v, NPFR c01896 = 8. The data in A, B, and C represent recordings from 7- to 14-day-old flies of both sexes (50:50) presented as means ± 95% confidence intervals. Two-way analysis of variance (ANOVA), non-significant (ns). (PDF 1331 kb) [file 12915_2017_374_MOESM2_ESM.pdf]

**A**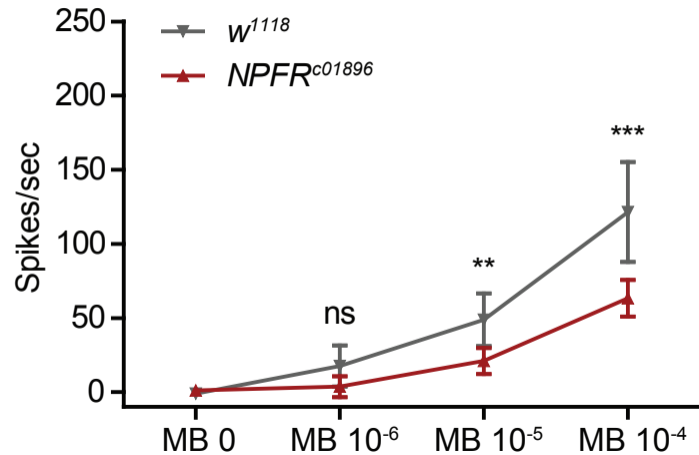**B**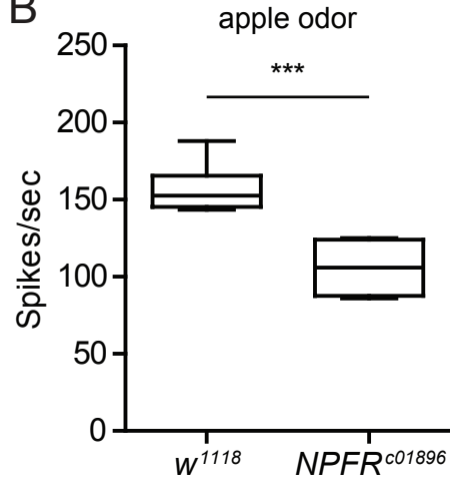

Supplement: Supplementary file 3 — NPFR loss of function reduces the responses of ab3A neurons to methyl butyrate and apple odor. A Peak odor-evoked activity of ab3A neurons from w 1118 and w 1118 ; NPFR c01896 mutant flies responding to solvent (paraffin oil, PO) or a major apple odor: methyl butyrate (MB, 10−6 v/v, 10−5 v/v, 10−4 v/v). The data in A represent recordings from 7- to 14-day-old flies of both sexes and are presented as means ± 95% confidence intervals. Two-way ANOVA, non-significant (ns), P < 0.01 (**), P < 0.001 (***). Replicate numbers for PO, w 1118 = 12; MB 10−6 v/v, w 1118 = 6; MB 10−5 v/v, w 1118 = 6; MB 10−4 v/v, w 1118 = 6; PO, NPFR c01896 = 12; MB 10−6 v/v, NPFR c01896 = 6; MB 10−5 v/v, NPFR c01896 = 6; MB 10−4 v/v, NPFR c01896 = 6. B Peak odor-evoked activity from ab3A neurons of 7- to 14-day-old flies of both sexes responding to apple skin odor. Boxplot whiskers indicate minimum and maximum values, one-way ANOVA, P < 0.001 (***). Replicate numbers for apple, w 1118 = 6; apple, NPFR c01896 = 7. (PDF 1298 kb) [file 12915_2017_374_MOESM3_ESM.pdf]

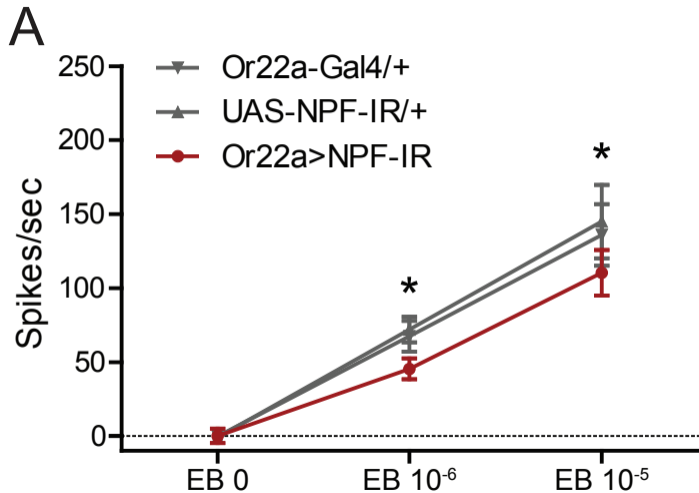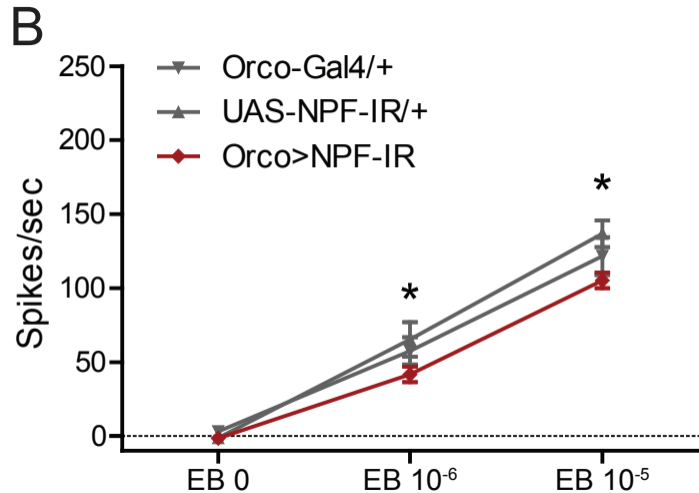

Supplement: Supplementary file 4 — OSN-specific knock-down of NPF also affects ab3A responses. A Peak odor-evoked activity of ab3A neurons from Or22a > NPF-IR flies and the appropriate heterozygous GAL4 and UAS controls (crossed to w 1118) responding to solvent (paraffin oil, PO) or ethyl butyrate (EB 10−6 or 10−5 v/v). Replicate numbers for PO, Or22a-GAL4+ = 16; EB 10−6 v/v, Or22a-GAL4/+ = 8; EB 10−5 v/v, Or22a-GAL4/+ = 8; PO, UAS-NPF-IR/+ = 12; EB 10−6 v/v, UAS-NPF-IR/+ = 6; EB 10−5 v/v, UAS-NPF-IR/+ = 6; PO, Or22a > NPF-IR = 12; EB 10−6 v/v, Or22a > NPF-IR = 6; EB 10−5 v/v, Or22a > NPF-IR = 6. B Peak odor-evoked activity of ab3A neurons from Orco > NPF-IR flies and the appropriate heterozygous GAL4 and UAS controls (crossed to w 1118) responding to solvent (paraffin oil, PO) or ethyl butyrate (EB 10−6 or 10−5 v/v). These data in A and B are presented as means ± 95% confidence intervals. Two-way ANOVA, P < 0.05 (*). Replicate numbers for PO, Orco-GAL4/+ = 12; EB 10−6 v/v, Orco-GAL4/+ = 6; EB 10−5 v/v, Or22a-GAL4/+ = 6; PO, UAS-NPF-IR/+ = 12; EB 10−6 v/v, UAS-NPF-IR/+ = 6; EB 10−5 v/v, UAS-NPF-IR/+ = 6; PO, Or22a > NPF-IR = 16; EB 10−6 v/v, Or22a > NPF-IR = 8; EB 10−5 v/v, Or22a > NPF-IR = 8. (PDF 1289 kb) [file 12915_2017_374_MOESM4_ESM.pdf]

NPFR-RD isoform

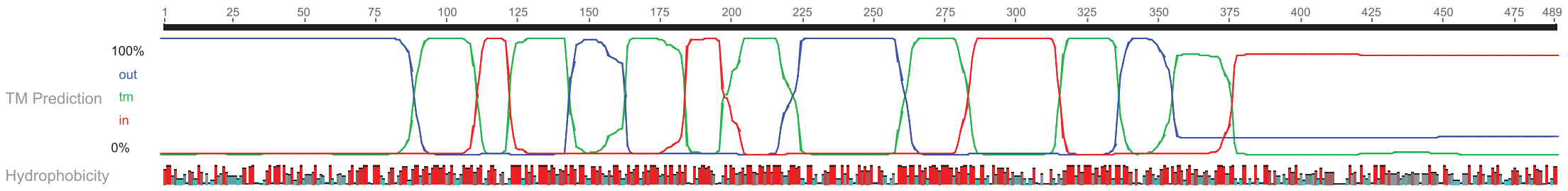

NPFR-RA isoform

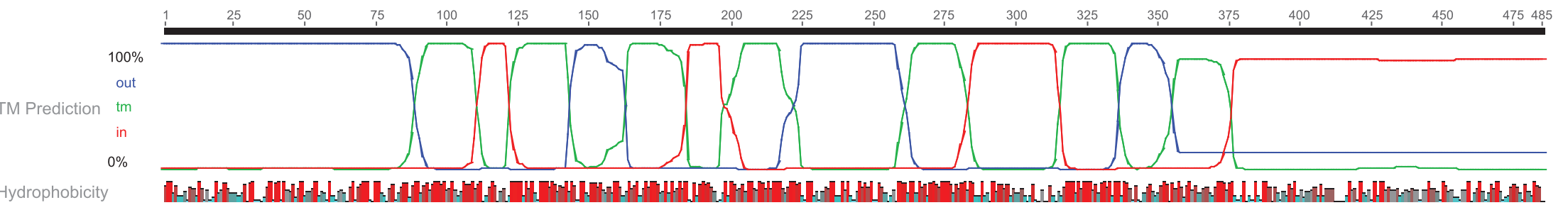

NPFR-RB isoform

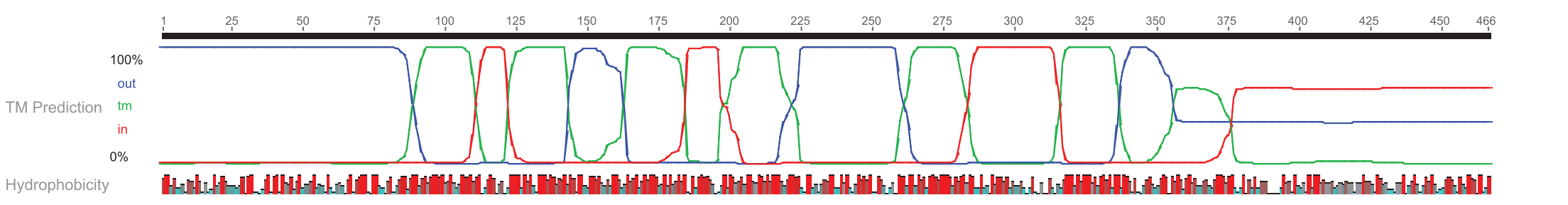

NPFR-RC isoform

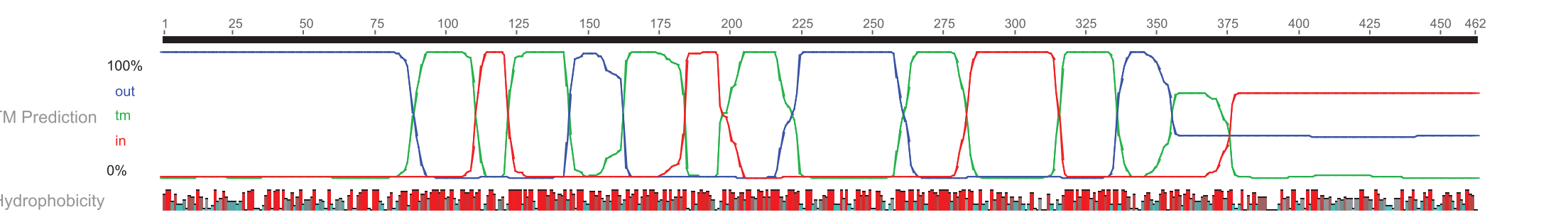

Supplement: Supplementary file 5 — Hydrophobicity plots for the various NPFR isoforms. Hydrophobicity plots and membrane topology predictions for the NPFR-RD, RA, RB, and RC isoforms. (PDF 1821 kb) [file 12915_2017_374_MOESM5_ESM.pdf]

A

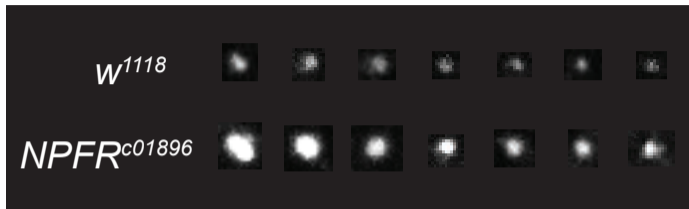

B

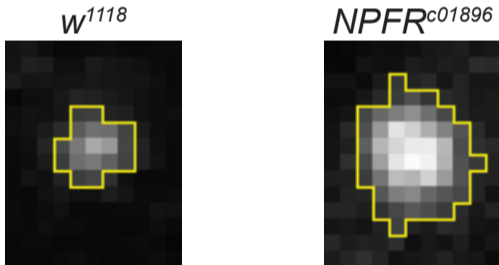

Supplement: Supplementary file 6 — Staining OR22a-positive puncta. A The seven puncta with the highest signal intensities from w 1118 and NPFR c01896 antennal sections. B Examples of region of interest selection by ImageJ where only pixels with intensity scores above 50 are included (yellow boundary). (PDF 1353 kb). [file 12915_2017_374_MOESM6_ESM.pdf]
